# Altering MYC phosphorylation in the epidermis increases the stem cell population and contributes to the development, progression, and metastasis of squamous cell carcinoma

**DOI:** 10.1038/s41389-020-00261-3

**Published:** 2020-09-07

**Authors:** Xiaoyan Wang, Ellen M. Langer, Colin J. Daniel, Mahnaz Janghorban, Vivian Wu, Xiao-Jing Wang, Rosalie C. Sears

**Affiliations:** 1 Department of Molecular and Medical Genetics, Oregon Health & Science University, Portland, OR USA; 2 Knight Cancer Institute, Oregon Health & Science University, Portland, OR USA; 3 Department of Otolaryngology-HNS, Henry Ford Health System, Detroit, MI USA; 4 Department of Pathology, University of Colorado Denver Anschutz Medical Campus, Aurora, CO USA; 5 Veterans Affairs Medical Center, VA Eastern Colorado Health Care System, Aurora, CO USA

**Keywords:** Squamous cell carcinoma, Cancer stem cells, Stem cells

## Abstract

cMYC (MYC) is a potent oncoprotein that is subject to post-translational modifications that affect its stability and activity. Here, we show that Serine 62 phosphorylation, which increases MYC stability and oncogenic activity, is elevated while Threonine 58 phosphorylation, which targets MYC for degradation, is decreased in squamous cell carcinoma (SCC). The oncogenic role of MYC in the development of SCC is unclear since studies have shown in normal skin that wild-type MYC overexpression can drive loss of stem cells and epidermal differentiation. To investigate whether and how altered MYC phosphorylation might affect SCC development, progression, and metastasis, we generated mice with inducible expression of MYC^WT^ or MYC^T58A^ in the basal layer of the skin epidermis. In the T58A mutant, MYC is stabilized with constitutive S62 phosphorylation. When challenged with DMBA/TPA-mediated carcinogenesis, MYC^T58A^ mice had accelerated development of papillomas, increased conversion to malignant lesions, and increased metastasis as compared to MYC^WT^ mice. In addition, MYC^T58A^-driven SCC displayed stem cell gene expression not observed with MYC^WT^, including increased expression of *Lgr6, Sox2*, and *CD34*. In support of MYC^T58A^ enhancing stem cell phenotypes, its expression was associated with an increased number of BrdU long-term label-retaining cells, increased CD34 expression in hair follicles, and increased colony formation from neonatal keratinocytes. Together, these results indicate that altering MYC phosphorylation changes its oncogenic activity—instead of diminishing establishment and/or maintenance of epidermal stem cell populations like wild-type MYC, pS62-MYC enhances these populations and, under carcinogenic conditions, pS62-MYC expression results in aggressive tumor phenotypes.

## Introduction

The skin epidermis is a stratified epithelium composed primarily of keratinocytes whose proliferation and differentiation must be tightly regulated in order to maintain skin homeostasis and respond effectively to injury^1^. Multipotent epidermal stem cells are slow-cycling cells with unlimited regenerative potential that reside within the basal layer of the epidermis and bulge region of the hair follicle, and they function to ensure that the epidermis is maintained during adult life. They give rise to transit-amplifying cells that undergo a small number of divisions before terminally differentiating to form the interfollicular epidermis (IFE), hair follicles, and sebaceous glands^2–5^.

MYC is a potent transcription factor that can regulate diverse cellular phenotypes including cell proliferation, apoptosis, maintenance of pluripotency, cellular reprogramming, and differentiation. For example, cMYC has been shown to be critical in the maintenance of pluripotency in embryonic stem cells and to inhibit differentiation of a variety of cell types when ectopically expressed^6–8^. In the epidermis, however, ectopic MYC expression has been shown, in human keratinocytes as well as mouse models, to promote differentiation by driving epidermal stem cells into transit-amplifying cells^9–11^. In apparent contrast, MYC deletion from the epidermis also depleted the stem cell pool, initiating differentiation prematurely and leading to thinning of the IFE as well as impaired wound healing^12^. Thus, the role of MYC in the epidermis is complex, and tight regulation of MYC function is likely important for normal skin homeostasis. Several mouse models have been generated to further examine the effects on tumor development following MYC expression in basal (which includes the stem cell compartment of the IFE, sebaceous gland and hair follicle) or suprabasal (terminally differentiating) keratinocytes. Results from these models indicate that driving MYC in the basal compartment results in hair loss, development of spontaneous wounds, and an increase in sebaceous gland size and number at the expense of hair follicles^10,13^, whereas driving MYC in the suprabasal epidermis produced papillomas that rarely progressed^14,15^. Thus, MYC overexpression appears to have varying effects depending on the cell type used in these models^16^.

Because MYC potently regulates most transformed cell phenotypes, MYC expression is normally tightly regulated through multiple mechanisms. One such mechanism involves sequential and interdependent phosphorylation of two conserved residues, Serine 62 (S62) and Threonine 58 (T58), that help regulate MYC turnover after cell growth stimulation^17^. S62 is phosphorylated by ERK1/2, JNK, CDK2, or CDK5 in response to growth stimulation, and this modification increases MYC protein stability^18–20^. Phosphorylation at S62 (pS62) also serves as a priming site for GSK3 to phosphorylate T58, which then enhances MYC degradation. Efficient MYC degradation requires dephosphorylation of S62 by Protein Phosphatase 2A (PP2A) and ubiquitination of pT58-MYC by the E3 ubiquitin ligase SCF-FBW7, ultimately leading to proteosome-mediated degradation of MYC^21^. The scaffold protein Axin1 enhances MYC degradation by coordinating formation of this MYC destruction complex^22^. Mutations in PP2A subunits, *FBW7*, and *AXIN1* have been identified in human cancers^23,24^, suggesting deregulation of this MYC degradation pathway. Moreover, elevated S62 and decreased T58 phosphorylation of MYC is found in several human cancer types^22,25,26^. Importantly, recent data indicates that pS62-MYC is targeted by the PIN1 proline isomerase that activates MYC to drive a subset of oncogenic target genes involved in cell proliferation and migration^27,28^.

In the current study, we examined pS62-MYC and pT58-MYC expression in human squamous cell carcinoma (SCC) and found that the stable, active pS62-MYC is highly expressed in human skin cancers as compared to normal skin. Given the divergent activity of MYC in skin on stem cell maintenance, epidermal differentiation, and tumorigenesis, we asked whether regulating MYC phosphorylation can affect the activity of MYC on stem cell maintenance or tumor development. We utilized our inducible *Rosa26-Myc*^*WT*^ and *Rosa26-Myc*^*T58A*^ knock-in mice to determine the effects of MYC^T58A^ versus MYC^WT^ expression in the epidermal basal layer of the skin, and found that expression of MYC^T58A^, which remains constitutively phosphorylated at S62, in skin epidermis accelerates tumorigenesis, enhances malignant conversion, and increases metastasis in a two-stage carcinogenesis protocol. We show that MYC^T58A^ expression is associated with increased proliferation in the epidermis adjacent to carcinogen-induced papillomas as well as decreased apoptosis in the hair follicles compared to MYC^WT^. Finally, we show that MYC^T58A^ expression results in an increased epidermal stem cell population through BrdU retention assays, CD34 staining, and keratinocyte rapid attachment assays. Our data suggest that post-translational activation of MYC alters its activity to promote stem cell renewal and the progression of malignant transformation from the basal and stem cell compartments of the skin epidermis.

## Results

### pS62-MYC is increased and pT58-MYC is decreased in clinical SCC samples relative to normal skin

**Fig. 1:**
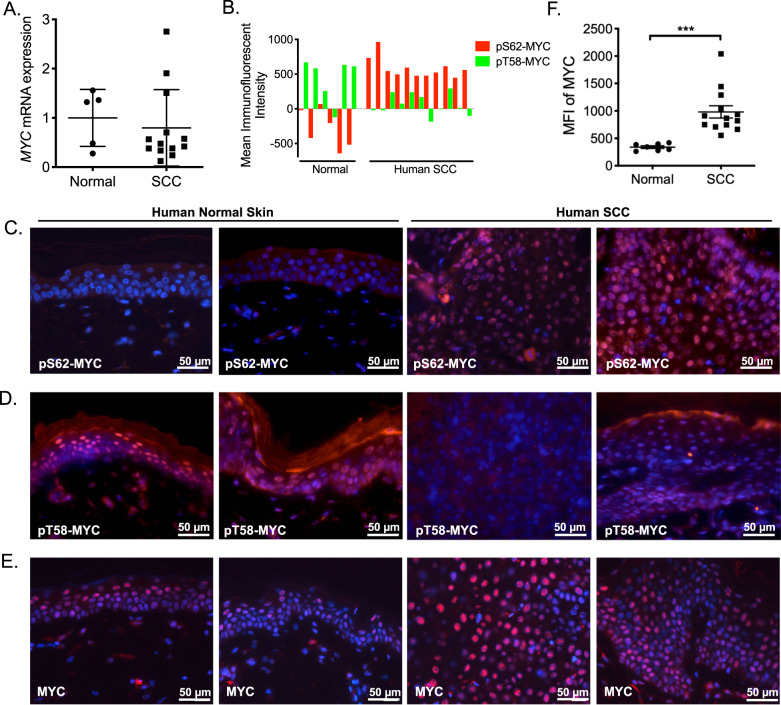
MYC phosphorylation at Serine 62 and Threonine 58 is altered in human squamous cell carcinomas (SCC). **a** Analysis of *MYC* mRNA expression by qRT-PCR in 13 human SCC samples and 5 adjacent skin tissues. **b** Immunofluorescence intensity of pS62-MYC and pT58-MYC was analyzed in 250 random cells for each patient sample using Open-Lab 5.5 software, with mean fluorescence intensity (MFI) displayed for 6 human normal skin and 11 human SCC samples. **c**–**e** Immunofluorescence staining of representative human normal skin and SCC samples quantified in (**b** and **f**) with (**c**) anti-pS62-MYC, (**d**) anti-pT58-MYC, and (**e**) anti-MYC. DAPI (blue) is nuclear counterstain. **f** Mean fluorescent intensity of MYC (**e**) was analyzed in 6 human normal skin and 11 human SCC samples. For each sample, ~50 cells were analyzed in each of five randomly selected areas, and an average MFI for each sample is shown. Significance was determined with an unpaired Student’s *t*-test, *p* < 0.001.

To examine whether changes in MYC expression occur in skin cancer, we first extracted RNA from 13 human SCC and 5 adjacent skin samples and performed qRT-PCR for *cMYC* expression. We did not find a significant difference in *cMYC* levels; *cMYC* mRNA was upregulated in only 2 of the 13 (15%) SCC samples as compared to adjacent normal skin (Fig. 1a). In our previous work, we observed that MYC was regulated at the protein level, with high levels of pS62-MYC and low levels of pT58-MYC in breast tumor cell lines and tumor tissue relative to non-transformed mammary cell lines and adjacent normal breast tissue, respectively^26,29,30^. To investigate whether MYC is similarly post-translationally regulated in skin cancer, we obtained formalin fixed paraffin embedded (FFPE) tissue from eleven human SCC samples and six adjacent normal skin samples and analyzed these by immunofluorescence (IF) for pS62- and pT58-MYC expression using phosphorylation-specific antibodies^26^. We found high expression of pS62-MYC in all of the SCC samples relative to the normal skin (Fig. 1b, c). In contrast, the level of pT58-MYC was reduced in SCC relative to adjacent normal skin (Fig. 1b, d). Consistent with this change in phosphorylation status, the level of MYC expression was elevated in SCC relative to adjacent normal skin (Fig. 1e, f). These results indicate an upregulation of MYC protein and an altered ratio of pS62/pT58 consistent with a more stable and selectively transcriptionally active form of MYC in skin carcinoma, suggesting that post-translational regulation of MYC may contribute to the role for MYC in skin tumorigenesis.

### Expression of exogenous MYC^T58A^ in the skin epidermis and hair follicles accelerates papilloma formation and malignant transformation following DMBA/TPA-mediated carcinogenesis

**Fig. 2:**
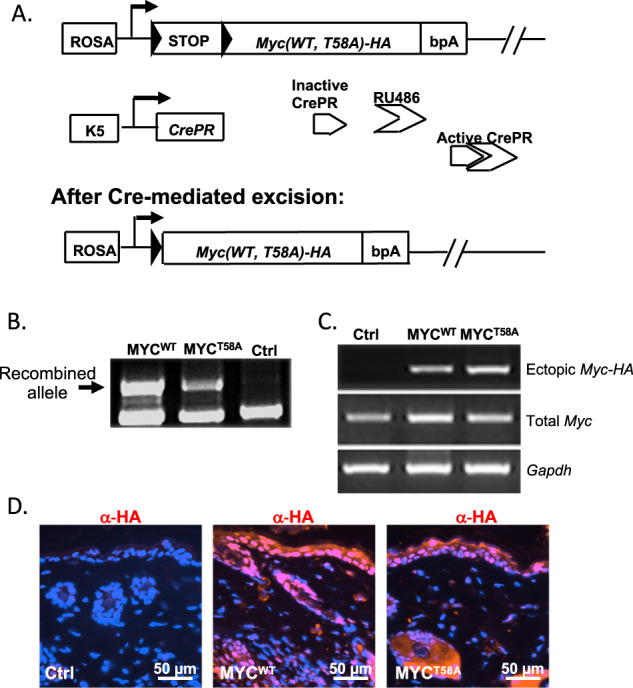
Generation and characterization of conditional knock-in transgenic mouse models of skin expressing MYC^WT^ or MYC^T58A^. **a** Knock-in strategy for conditional expression of MYC^WT^-HA and MYC^T58A^-HA. Rosa-Floxed-Stop (RFS)-*Myc* constructs are shown. Arrowheads indicate loxP sites. bpA is a transcription stop sequence. Inserted *cMyc* cDNAs have a C-terminal HA tag. The K5-*CrePR* transgene encodes a fusion protein consisting of Cre recombinase and a truncated progesterone receptor driven by the Keratin 5 (K5) promoter. Cre recombination allows ectopic *Myc*^*WT* or *T58A*^ expression driven from the ROSA promoter. **b** Recombined allele was detected in skin from MYC^WT^ and MYC^T58A^ mice after RU486 treatment. The control is RFS-*Myc*^*WT*^ without K5-*CrePR* after RU486 treatment. **c** PCR showing ectopic and total *Myc* mRNA expression in MYC^WT^ or MYC^T58A^ mice compared to the control (no Cre) mice 8 weeks after RU486 induction. **d** Representative images showing expression of ectopic MYC by HA staining in normal skin tissues of Ctrl, MYC^WT^, MYC^T58A^ mice 8 weeks after RU486 treatment.

To determine the role of MYC phosphorylation in skin, we utilized mice that have conditional expression of MYC^WT^ or MYC^T58A^ from the *Rosa26* locus (RFS-*Myc*^WT^ or RFS-*Myc*^T58A^)^31^. The MYC^T58A^ mutant imitates the phosphorylation ratio of S62 and T58 found in human SCC, maintaining high S62 phosphorylation because the lack of phosphorylation at T58 makes the protein resistant to PP2A-mediated dephosphorylation^32^. To drive MYC expression in the skin, we crossed the RFS-*Myc*^WT^ or RFS-*Myc*^T58A^ mice with Keratin 5 (K5)-*CrePR* transgenic mice, in which the K5 promoter drives expression of Cre recombinase fused to a truncated progesterone receptor in the basal layer of the epidermis^33^. Treatment with RU486 induces activity of Cre recombinase, driving deregulated expression of MYC^WT^ or MYC^T58A^ in the basal epidermis (Fig. 2a). We treated the backs of five-week old experimental K5-*CrePR*;RFS-*Myc*^WT^ (MYC^WT^) and K5-*CrePR*;RFS-*Myc*^T58A^ (MYC^T58A^) mice and control RFS-*Myc*^WT^ or RFS-*Myc*^T58A^ mice without K5-*CrePR* with RU486 for 5 days. PCR analysis of genomic DNA extracted from back skin of these mice at 2 months of age showed that the recombined allele was detected in the skin of MYC^WT^ and MYC^T58A^ mice, but not in the control mice (Fig. 2b). Initial RT-PCR analysis demonstrated expression of ectopic *cMyc*-*HA* mRNA in the MYC^WT^ and MYC^T58A^ skin samples (Fig. 2c). Immunofluorescent analysis showed that MYC-HA was present in the epidermis and hair follicles of the MYC^WT^ and MYC^T58A^ mice (Fig. 2d). These mice were monitored for 20 months, and we did not observe development of any skin lesions indicating that this modest expression of ectopic MYC^WT^ or MYC^T58A^ from the *Rosa26* locus in adult skin is not oncogenic.

**Fig. 3:**
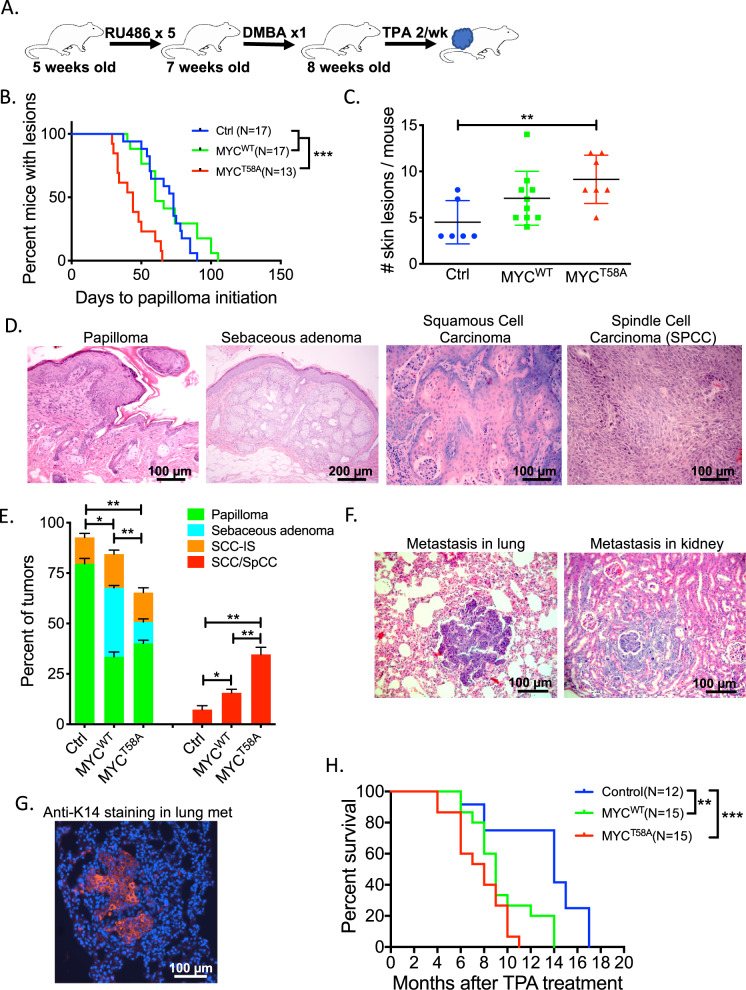
Increased tumor initiation and promotion in MYC^T58A^ mice. **a** Schematic of treatment strategy for papilloma initiation after DMBA/TPA treatment. Age matched mice of the indicated strains were treated with DMBA/TPA according to the two-step chemical carcinogenesis assay. **b** Kaplan–Meier curve showing days to papilloma initiation since the first TPA treatment of skin from mice of each indicated strain. Significance was determined with a Log-rank test, ***p < 0.001. **c** Number of skin lesions per mouse after DMBA/TPA treatment. Mean ± SD is shown, significance was determined by one-way ANOVA, followed by Tukey’s multiple comparison test, **p < 0.01. **d** Representative H&E staining of different lesions formed following DMBA/TPA treatment including papilloma, sebaceous adenoma, squamous cell carcinoma (SCC), spindle-cell carcinoma (SpCC). Scale bars are as indicated. **e** Quantification of different lesion types at 26–28 weeks after DMBA/TPA treatment. *n* = 5 mice per genotype, and percent of each lesion type is graphed ±SD. SCC-IS indicates in situ lesions. Student’s *t*-test was used to compare total number between genotypes of benign or malignant lesions, *p < 0.05, **p < 0.01. **f** Representative images of SCC metastasis to lung and kidney. Scale bars = 100 µm. **g** SCC metastasis in lung was confirmed by immunofluorescence using anti-K14 (red). **h**. Survival curves for each strain following DMBA/TPA treatment (n as indicated). Significance was determined with a Log-rank test. **p < 0.01, ***p < 0.001.

We next utilized a classic two-stage chemical carcinogenesis model, where initial treatment with 7,12-dimenthylbenz[a]anthracene (DMBA) induces mutations in the epidermis and is followed by repeated administration of 12-o-tetradecanoylphorbol-13-acetate (TPA) that elicits an inflammatory response to mediate further transformation, resulting in papilloma development^34,35^. To address the consequences of altering MYC phosphorylation in this model, we treated the MYC^WT^, MYC^T58A^, and control mice with DMBA and TPA according to the two-step chemical carcinogenesis protocol (see Fig. 3a). Two weeks prior to DMBA treatment, MYC^WT^ or MYC^T58A^ expression was induced as above by painting RU486 on the back skin. A single dose of DMBA was followed by administration of TPA twice per week until study endpoint. Expression of ectopic HA-tagged MYC^WT^ and MYC^T58A^ was confirmed by immunofluorescence in skin hyperplasia and papillomas from MYC^WT^ and MYC^T58A^ mice (Supplemental Fig. S1a). Expression of ectopic MYC^WT^ or MYC^T58A^ in papillomas resulted in decreased expression of endogenous *cMyc* mRNA (Supplemental Fig. S1b), consistent with other models where ectopic MYC has been shown to autoregulate and suppress its own promoter^36,37^. This, together with the relatively weak ROSA promoter, resulted in total *Myc* mRNA levels that were only modestly higher in papillomas from MYC^WT^ and MYC^T58A^ mice compared to control mice papillomas (Supplemental Fig. S1c). Consistent with previous studies, pS62-MYC staining appeared higher with expression of MYC^T58A^ (Supplemental Fig. S1d)^26,29,30^.

The MYC^T58A^ mice were found to develop papillomas on average 45 days after the DMBA/TPA treatment was initiated, which was early as compared with the MYC^WT^ and control mice, which developed papillomas on average 69 and 68 days after DMBA/TPA treatment, respectively (Fig. 3b and Table 1). In addition, the MYC^T58A^ mice developed an increased number of skin lesions, averaging about nine lesions per mouse. The MYC^WT^ mice developed an average of about seven skin lesions per mouse compared with the control mice which averaged 4.5 lesions per mouse (Fig. 3c). To determine whether expression of MYC^T58A^ not only affected the timing and number of lesions, but also the malignancy, we performed H&E staining of skin tumors from mice of each genotype treated with RU486 and DMBA/TPA and taken down at 26–28 weeks. Using histopathological analysis, we quantified the prevalence of benign (papilloma, sebaceous adenoma, or in situ SCC) and malignant (SCC or spindle-cell carcinoma (SpCC)) lesions for the different genotypes (Fig. 3d, e). This analysis revealed that expressing MYC^T58A^ in the skin dramatically enhanced the malignant potential of the lesions, with 32.4% incidence of SCC and SpCC as compared to 15.9% within the MYC^WT^ mice and 11.1% within the control mice. Finally, when tumors were allowed to grow to the study endpoint, expression of MYC^T58A^ was found to dramatically enhance metastatic properties. The MYC^T58A^ mice had a 57% incidence of metastasis, with metastatic foci evident on the surface of liver and lungs, as well as in kidney, brain, and mammary glands. In comparison, the MYC^WT^ and control mice had 13% and 16% incidence of metastasis, respectively (Fig. 3f, g and Table 1). Importantly, the MYC^T58A^ mice also had a shortened median survival to study endpoint of ~7.6 months compared to the MYC^WT^ with a median survival of 9.7 months and the control mice with a median survival of 13.4 months (Fig. 3h and Table 1).

**Table 1:** Summary of phenotypes from DMBA/TPA experimental mice.

Genotype	Papilloma initiation (days)	Incidence of metastasis (%)	Survival (months)
Control (*N* = 8)	68 (±5)	16.70%	13.4
Myc^WT^ (*N* = 14)	69 (±8)	13.30%	9.7
Myc^T58A^ (*N* = 10)	45 (±5)	57%	7.6

### MYC^T58A^ expression reduces apoptotic potential and enhances proliferative activity relative to MYC^WT^ in DMBA/TPA-treated epidermis

**Fig. 4:**
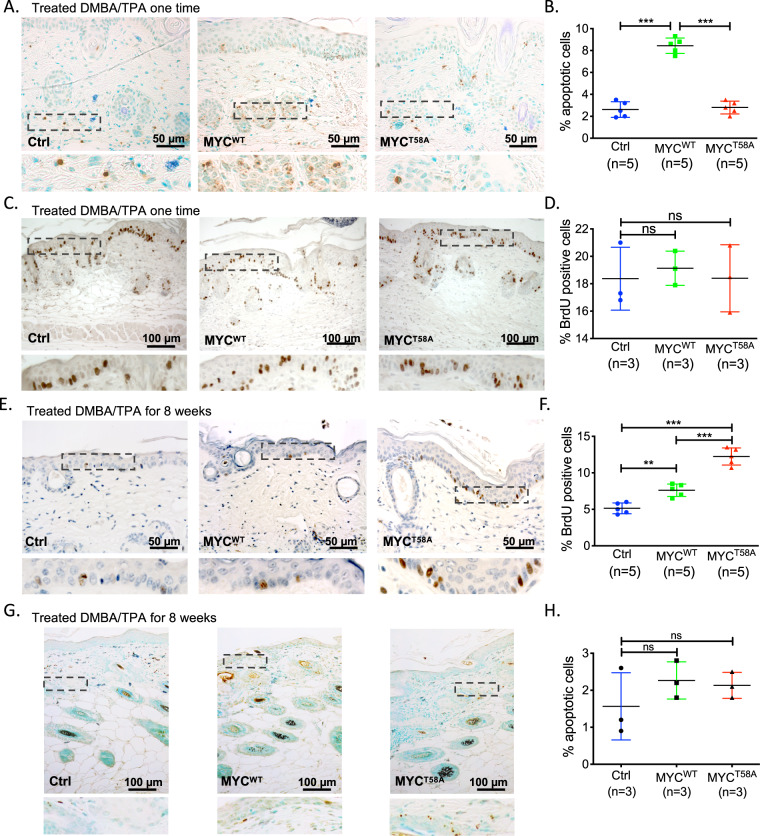
MYC^T58A^ expression results in increased proliferation and reduced apoptosis after DMBA/TPA chemical carcinogenesis treatment. **a**, **b** TUNEL staining (**a**) and quantitation (**b**) to assess apoptosis in the epidermis and hair follicle of mice 72 h post DMBA/TPA one-time treatment. Scale bar = 50 µm. Shown is mean and SD of 5 mice per genotype. *p*-value is from a one-way ANOVA, followed by Tukey’s multiple comparison test, ***p < 0.001. **c**, **d** BrdU staining (**c**) and quantitation (**d**) to assess proliferation in the epidermis and hair follicle of mice 48 h post DMBA/TPA one-time treatment. Scale bar = 100 µm. Shown is mean and SD of 3 mice per genotype. *p*-value is from a one-way ANOVA, followed by Tukey’s multiple comparison test, ns = not significant. **e**, **f** BrdU staining (**e**) and quantitation (**f**) in the epidermis adjacent to the skin lesions after 8 weeks of DMBA/TPA treatment of mice from each genotype. Scale bar = 50 µm. Shown is mean and SD of 5 mice per genotype. *p*-value is from a one-way ANOVA, followed by Tukey’s multiple comparison test, **p < 0.01, ***p < 0.001. **g**, **h** TUNEL staining (**g**) and quantitation (**h**) to assess apoptosis in the epidermis and hair follicle of mice 8 weeks post DMBA/TPA treatment. Scale bar = 100 µm. Shown is mean and SD of 3 mice per genotype. *p*-value is from a one-way ANOVA, followed by Tukey’s multiple comparison test, ns = not significant.

The increased number and malignancy of lesions following DMBA/TPA treatment with MYC^T58A^ expression suggested that epidermal apoptosis might be impaired and/or proliferation might be enhanced. To test whether apoptosis was impaired, we performed the two-step DMBA/TPA chemical carcinogenesis experiment and analyzed apoptosis by TUNEL assay in the skin 72 h after the first TPA treatment. We found a dramatic increase in apoptosis in the epidermis and hair follicles from the MYC^WT^ mice, which is consistent with the role of MYC as a potent inducer of apoptosis under growth-limiting conditions^38^. In contrast, apoptosis in the skin of MYC^T58A^ mice was substantially reduced relative to MYC^WT^ and comparable to that in control mice (Fig. 4a, b), which is consistent with reports that expressing MYC^T58A^ does not induce apoptosis like MYC^WT^ and can suppress apoptosis under conditions like mammary gland involution^31,39^. We also assessed proliferation by injecting each mouse with 100 μg BrdU 2 h prior to collecting skin samples 48 h after DMBA/TPA treatment. We found no difference in proliferation between the samples at this early time point (Fig. 4c, d). To assess proliferation and apoptosis in the skin at later time points, we again performed the two-step DMBA/TPA chemical carcinogenesis experiment, treating with TPA for 8 weeks. For proliferation analysis, 2 h prior to collecting skin samples, each mouse was injected with BrdU. We observed consistent, marked elevation of BrdU incorporation in the epidermis adjacent to lesions from MYC^T58A^ mice when compared with either the MYC^WT^ or control mice (Fig. 4e, f), indicating that expression of MYC^T58A^ supported sustained proliferation in the adjacent epidermis. Unlike early time points, we found no difference in apoptosis rates in the skin after 8 weeks of treatment (Fig. 4g, h).

### MYC^T58A^ expression drives enhanced progenitor cell characteristics in both normal epidermis and in tumors

**Fig. 5:**
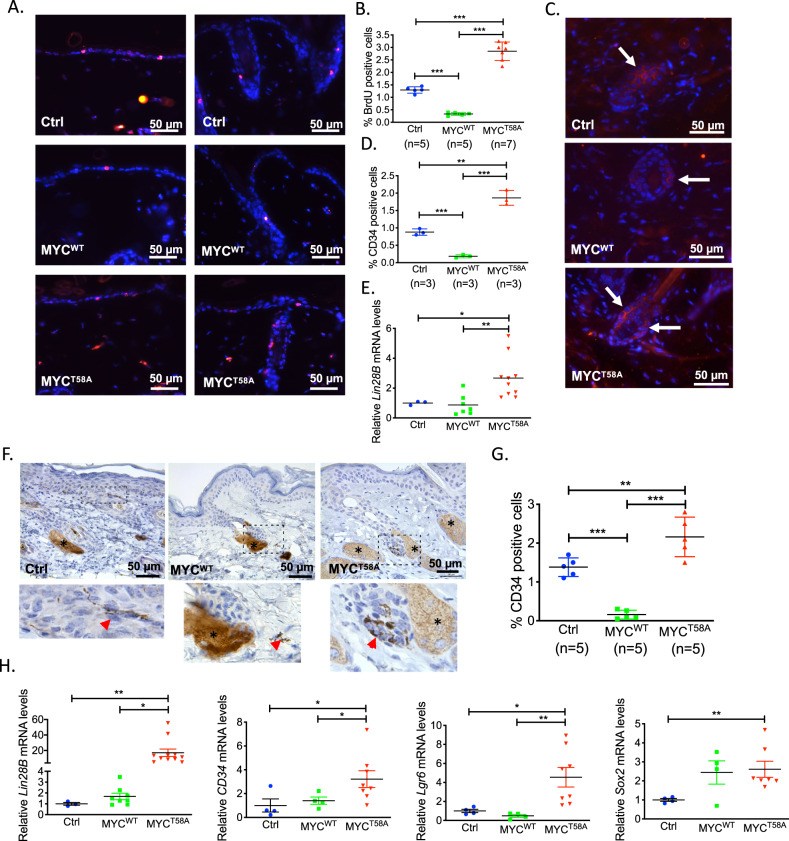
Increased stem cell populations in skin of MYC^T58A^ mice. **a**, **b** Long-term label-retaining cell analysis in control, MYC^WT^, or MYC^T58A^ mice. Retention of BrdU was assessed 75 days after injection through immunofluorescent staining for BrdU (red) shown in (**a**) and quantification of percent of cells positive for BrdU shown in (**b**). *p*-value is from a one-way ANOVA, followed by Tukey’s multiple comparison test, ***p < 0.001. **c** Immunofluorescence images of skin from the long-term label-retaining analysis stained with anti-CD34 (red). White arrows indicate positive cells in hair follicles. DAPI (blue) is nuclear counterstain. **d** Quantitation of CD34 staining from (c). Significance was determined by one-way ANOVA, followed by Tukey’s multiple comparison test, **p < 0.01, ***p < 0.001. **e** qRT-PCR for *Lin28B* expression in skin of mice used in long-term label-retaining analysis (normal skin). *p*-value is from a one-way ANOVA, followed by Tukey’s multiple comparison test, *p < 0.05, **p < 0.01. **f** Immunohistochemistry for CD34 staining in hair follicles from the hyperplastic skin of control, MYC^WT^, or MYC^T58A^ mice 4 months after DMBA/TPA treatment. Scale bar = 50 µm. Insets are shown below. Red arrowheads indicate CD34 positive cells. Nonspecific staining in the sebaceous gland is indicated by asterisks. **g** Quantification of CD34 staining from mice as in (**f**). Shown is mean and SD of 5 mice per genotype. *p*-value is from a one-way ANOVA, followed by Tukey’s multiple comparison test, *p < 0.05, ***p < 0.001. **h** qRT-PCR for expression of stem cell marker or signaling pathway genes, as indicated, in hyperplastic epidermis from DMBA/TPA-treated control, MYC^WT^, or MYC^T58A^ mice. *p*-value is from a two-tailed Welch’s *t*-test. *p < 0.05, **p < 0.01, ***p < 0.001.

MYC has divergent roles in the maintenance of stem cell populations^9,40,41^. In the skin, it can drive keratinocyte stem cell differentiation into transit amplifying cells^10^. To determine whether an alteration in MYC phosphorylation can differentiate between MYC activities in stem cells we analyzed the number of stem cells between genotypes in our study. We performed a BrdU long-term label-retaining experiment^10^ to measure stem cell populations in the MYC^WT^, MYC^T58A^, and control mice. First, we treated the back skin of 5-day-old pups with RU486 for five days and then injected the 10-day-old pups with four doses of BrdU over 48 h. Skin samples were collected 75 days after labeling, and the BrdU label-retaining cells (LRCs) after this 75-day chase period are considered epidermal stem cells defined by long life and low replication index. The percent of label-retaining cells was found to be 1.3% in the control mice, 0.23% in the MYC^WT^ mice, and 2.85% in the MYC^T58A^ mice (Fig. 5a, b). These results indicate that MYC^WT^ and MYC^T58A^ function differently in this tissue, resulting in decreased or increased numbers of stem cells, respectively. To further support this result, we performed IF for CD34, a marker of the infrequently cycling and label-retaining cells in the hair follicle bulge^42,43^, on the skin from these mice. Again, we found a decrease in the number of CD34^+^ stem cells in the MYC^WT^ mice and an increase in the number of CD34^+^ stem cells in the MYC^T58A^ mice as compared to the controls (Fig. 5c, d). We also performed qRT-PCR analysis for *Lin28B*, a MYC target gene that regulates stem cell self-renewal and has previously been shown to enhance the generation of induced pluripotent stem cells^44,45^. In our mice, we found that MYC^T58A^ expression specifically was associated with the upregulation of *Lin28B* in the normal adjacent skin (Fig. 5e). Together, these data suggest that MYC^WT^ decreases, while MYC^T58A^ increases the number of epidermal stem cells. These results are consistent with previous reports that MYC^WT^ drives epidermal stem cells to differentiate^9,11^, but also extend our understanding by showing that MYC^T58A^ is functionally distinct from MYC^WT^ as evidenced by its ability to increase the epidermal stem cell population.

To determine whether the MYC^T58A^-driven tumors also manifest a stem/progenitor cell phenotype, we performed immunohistochemical analysis with anti-CD34 in the hyperplastic epidermis from DMBA/TPA, 8-week treated MYC^T58A^ and MYC^WT^ mice compared to the control mice. A similar trend of decreased numbers of CD34 positive cells in the MYC^WT^ mice and increased numbers in the MYC^T58A^ mice was detected in the hyperplastic epidermis and hair follicles following the two-step carcinogenesis (Fig. 5f, g). To confirm increased stem cell gene expression in these hyperplastic regions, we also assessed the mRNA level of *Lin28B*, *CD34*, *Lgr6*, and *Sox2*, which can serve as markers for stem-like tumor cells in skin squamous cell carcinoma^46–49^. We found *Lin28B*, *Lgr6, CD34*, and *Sox2* mRNA expression significantly elevated specifically in the hyperplastic skin from MYC^T58A^ mice (Fig. 5h). *Sox2* expression was upregulated in some of the MYC^WT^ tissues as well. Together, these results demonstrate that MYC^T58A^ functions distinctly from MYC^WT^ to establish or maintain epidermal stem cell populations in both normal skin and induced skin tumors.

Since MYC^T58A^ expression drove increased expression of the stem cell markers *Lin28B*, *Lgr6*, and *Sox2* in this model, we also asked if the expression of these genes correlated in human squamous cell carcinomas. We chose to query head and neck squamous cell carcinoma (HNSCC) because the human cutaneous SCC datasets available had fewer samples and no RNA-seq information available. We confirmed by IF that HNSCC samples obtained from the OHSU Biolibrary, similar to SCC samples, showed upregulation of pS62-MYC and downregulation of pT58-MYC in tumors as compared to adjacent mucosa (Supplemental Fig. S1a, b). We then analyzed the gene amplification and mRNA upregulation of *MYC* in HNSCC (*N* = 279) as profiled by TCGA and queried on cBioPortal (HNSCC: Cancer Genome Atlas Network^50^, SCM: TCGA, PanCancer Atlas). The data showed that *MYC* is amplified or upregulated in 20.8% of HNSCC (Supplemental Fig. S2c). We next queried the provisional HNSCC TCGA dataset in cBioPortal in order to include MYC protein levels in the analysis. We assessed co-occurrence of high MYC with stem cell markers *SOX2*, *LGR6*, and *LIN28B* and found significant co-occurrence of upregulated MYC with *SOX2*, *LGR6*, and *LIN28B* in these tumors (Supplemental Fig. S2d, e).

### MYC^T58A^ expression increases stem cell phenotypes in primary keratinocytes

**Fig. 6:**
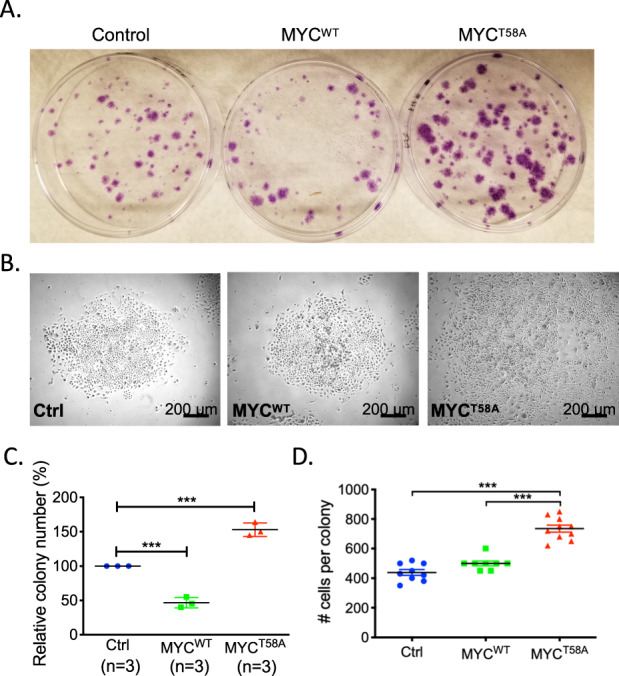
MYC^T58A^ expression increased stem cell phenotypes in primary keratinocytes. **a**, **b** Representative images of crystal violet stained plates (**a**) or phase contrast images (**b**) of primary keratinocyte colony after 10 days of culture from the rapid attachment assay of indicated genotype primary keratinocytes. **c**, **d** Quantitation of the number of colonies (**c**) and the number of cells per colony (**d**) in the rapid attachment assay. Data from each genotype includes keratinocytes pooled from four pups. *p*-values are from a one-way ANOVA, followed by Tukey’s multiple comparison test, ***p < 0.001.

Previous studies have suggested that in mouse skin ~10% of the basal cells are stem cells, and that these can be identified based on their ability to rapidly adhere to dishes in culture^51^. To further examine the effects of MYC^T58A^ expression in skin cells, we performed an in vitro rapid attachment assay with keratinocytes isolated from P3 neonatal mice (mice had been induced with RU486 three times since birth). Consistent with an increased number of LRCs in the MYC^T58A^ mice, we observed 50% more colonies formed after 10 days with MYC^T58A^ expressing keratinocytes. Conversely, MYC^WT^ expressing keratinocytes formed 50% fewer colonies than the control (Fig. 6a–c). In addition, the colonies from MYC^T58A^ mice had a more irregular shape and contained significantly more cells per colony when compared to those from either the control mice or the MYC^WT^ mice (Fig. 6a, b, d). Taken together with the in vivo data, these results support a role for altered MYC phosphorylation (loss of T58 and gain of S62 phosphorylation) in driving stem cell phenotypes, which likely contributes to tumorigenesis and cancer aggressiveness.

## Discussion

Mounting evidence indicates that the proto-oncogene MYC is overexpressed in a variety of human malignancies. In cancer, the activity of the MYC transcriptional network is frequently deregulated, contributing to the initiation and maintenance of disease. MYC levels can be elevated through gene amplification, transcriptional^52,53^ and post-transcriptional (e.g., FBXW7 ubiquitin ligase and RAS/MAPK pathway)^21,54,55^ mechanisms. Pan-cancer analyses of at least 12 cancer types estimated the frequency of *MYC* amplification at ~14%^56–58^. This is consistent with our observation here of 15% of clinical SCC samples showing increased *cMYC* mRNA levels. In contrast, MYC protein levels are generally elevated in a significantly higher percentage of tumors due to post-translational deregulation. For example, in SCC of the head and neck, overexpression of MYC by immunohistochemistry has been reported between 56.3 and 91%^59–61^.

While it is well-established that MYC protein is overexpressed in cancer, how post-translational modifications of MYC contribute to altered functions of MYC in the development, progression, and metastasis of SCC has not been reported. Previously, we showed that cMYC stability is in part controlled by sequential and interdependent phosphorylation at S62 and T58 and that these altered phosphorylation states also affect the activity and tumorigenic potential of cMYC^27,29,62^. In this study, we identify high levels of pS62-MYC and low levels of pT58-MYC in human SCC, suggesting that post-translational modifications regulating cMYC protein stability occur in the development of human SCC. In addition, we expressed the MYC^T58A^ mutant, which lacks T58 phosphorylation and has high S62 phosphorylation in the basal epidermis of mice to model the phosphorylation ratio of S62 and T58 found in human SCC. We found that MYC^T58A^ expressed in the skin epidermis and hair follicles could induce early papilloma development with DMBA/TPA-mediated carcinogenesis and could enhance malignant properties including increased metastatic foci in liver, lung, brain, kidney, and mammary gland. This was accompanied with increasing proliferation in the adjacent epidermis. These findings together validate that, in vivo, altered MYC phosphorylation at T58 and S62 affects cMYC’s oncogenic activity and contributes to the development, progression, and metastasis of SCC in two-stage skin carcinogenesis.

The epidermis is a self-renewing stratified epithelium. It has been reported that the proto-oncogene MYC stimulates keratinocyte proliferation and promotes differentiation of human epidermal stem cells^9^. Consistent with this, when we overexpress MYC^WT^ in the basal layer of the skin with K5-*CrePR*, we observe a decrease in the number of stem cells as evidenced by a decrease in the long-term label-retaining cell number, the number of cells positive for CD34, a marker of these stem cells^49^, and the number of colonies formed by isolated keratinocytes ex vivo. In contrast, our results show MYC^T58A^ expression in basal keratinocytes and hair follicles increased the number of stem cells, increasing the number of label-retaining cells, CD34 positive cells, and colony-forming keratinocytes.

We assessed markers of stem cells in the Control, MYC^WT^, or MYC^T58A^ expressing hyperplastic epidermis following DMBA/TPA treatment and found that MYC^T58A^ expression drives increased expression of *Lin28B*, *CD34, Lgr6*, and *Sox2*. Many reports have shown that CD34 is a stem cell marker in SCC, regulating tumor initiation and progression^49,63,64^, and a recent study demonstrated that LGR6 is a stem cell marker in skin SCC^46^. Both of these proteins mark normal stem cells as well. SOX2 has also been shown to be transcriptionally upregulated in skin SCC, it contributes to upregulation of LIN28, and it downregulates BMP4^47,65^. LIN28B has been shown to be a direct MYC target and functions to negatively regulate *Let-7* microRNAs to maintain a stem cell fate^66^. LIN28 has also been shown to be a pluripotency factor, capable of functioning with OCT4, NANOG, and SOX2 to reprogram human somatic fibroblasts to pluripotency^67^. In addition, LIN28 has been shown to contribute to transformation and its expression is correlated with poor prognosis in several human malignancies^44^. Together, the results of our study strongly indicate that MYC^T58A^, unlike MYC^WT^, supports the establishment and/or maintenance of epidermal stem cell populations associated with increased expression of multiple genes with known roles in stem cells and cancer. Further, the increased malignancy of the MYC^T58A^-expressing skin tumors in this model suggests that the tumors might arise from a progenitor/stem cell-like population.

In sum, this study utilizes a unique mouse model to reveal a role for T58 and S62 phosphorylation in the normal activity and oncogenic potential of cMYC in the skin. While expressing MYC^WT^ in the basal layer of the skin drove differentiation and proliferation of the skin, expressing MYC^T58A^ resulted in increased numbers of skin stem cells. In addition, MYC^T58A^ expressing epidermis was susceptible to increased oncogenesis and tumor progression following a chemical carcinogenesis treatment. MYC^T58A^ expression associated with increased cancer stem cell features that supported increased malignancy and distant metastasis. This model, therefore, provides a robust platform to further interrogate the role of MYC phosphorylation in cMYC function, which could impact strategies to therapeutically target cMYC.

## Methods

### Human tissue samples

De-identified human skin lesion and normal samples were provided by OHSU Department of Dermatology Molecular Profiling Tissue Repository (IRB#10071) and used for RNA extraction. De-identified formalin fixed paraffin embedded SCC and HNSCC tissues were obtained from the OHSU Knight Cancer Institute Biolibrary and used for immunostaining.

### Animals and animal care

Mouse care and experimental procedures were performed in accordance with established institutional guidance and approved by the Research Animal Care Committee of Oregon Health & Science University. Rosa-Floxed-Stop (RFS)-*Myc*^*WT*^ and RFS-*Myc*^*T58A*^ mice were previously described^31^. K5-*CrePR* mice were provided by Dr. Xiaojing Wang^33^ (University of Colorado Denver). Experimental mice carried homozygous RFS-*Myc*^*WT* or *T58A*^ and K5-*CrePR*.

### Reverse transcription-PCR (RT-PCR)

Total RNA from skin samples was extracted by TRIzol reagent (Invitrogen) according to manufacturer’s protocol, treated with DNaseI, and then purified over an RNeasy mini column (Qiagen). cDNA was made from DNaseI-treated RNA using High Capacity Reverse Transcription kit (Applied Biosystems) according to manufacturer’s protocol with random primers. Qualitative RT-PCR was performed to confirm expression of ectopic and total *MYC* in the mouse model. Quantitative RT-PCR analysis was performed on a StepOne qRT-PCR machine (Applied Biosystems) according to manufacturer’s preset PCR cycle conditions. Taqman primers were used for assessing human *MYC* (Hs00905030_m1). All other primers used in this study are listed in Table 2. *TBP* primers were used as controls to normalize expression level of each gene to *TBP* using the ΔΔCT method.

**Table 2:** Primer sequences for RT-PCR.

Gene	Forward	Reverse
Total *Myc*	GCCCCTAGTGCTGCATGAG	CCACAGACACCACATCAATTTCTT
murine *Myc* (endogenous)	CCCCAAGGTAGTGATCCTCA	AATTCCAGCGCATCAGTTAT
murine *Lin28B*	GTCTCACGGGTTTCGATTGAG	GTGATGTCCTATGTTTTGCCAAAT
murine *CD34*	GAAGTCCAGCCTGCCATCTAT	CAGCCTCAGCCTCCTCCTTTTCA
murine *Lgr6*	ATCATGCTGTCCGCTGACTG	ACTGAGGTCTAGGTAAGCCGT
murine *Sox2*	TGCTGCCTCTTTAAGACTAGGG	TCGGGCTCCAAACTTCTCT
murine *BMP4*	TGAGCTCCTGCGGGACTT	CGGCGCAGCCCAAAC
murine *TBP*	CTGGAATTGTACCGCAGCTT	TCCGTGGCTCTCTTATTCTCA

### Antibodies

Anti-HA-11 (G036, Applied Biological Material) 1:1000; anti-cMYC Y69 (ab32072, Abcam) 1:1000; anti-cMYC (4B12, made in house and recently described in *Methods Mol Biol Clifton NJ* (in press)) 1:500; anti-Keratin 14 (PRB-155p, Covance) 1:1000; anti-BrdU (MCA2060, AbD serotec) 1:200; anti-CD34 (ab8158 Abcam) 1:50 dilution; anti-cMYC-pS62 (Ab185656, Abcam) 1:500; anti-cMYC-pT58 (Y011034, Applied Biological Material) 1:500.

### Histological analysis and immunofluorescence

Mouse skin for histological analysis and immunofluorescence was collected and fixed in 10% formalin-neutral buffer. Samples were then embedded in paraffin, sectioned, and stained. Hematoxylin and eosin (H&E) staining was done according to standard protocols. Immunofluorescence on both mouse and human tissues was performed as previously described^31^. Images were taken with a Hamamatsu digital camera mounted on a fluorescence microscope. Immunofluorescence density was analyzed with Openlab 5.5 software using the Measure Density tool, with representative regions measured and averaged for each condition and graphed ±SD.

### DMBA/TPA-induced epidermal carcinogenesis procedure

Five-week-old mice were treated with 100 µl of 0.2 µg/µl RU486 for 5 consecutive days. The dorsal skin of mice was shaved and painted with DMBA (Sigma) at 100 µg per mouse 2 weeks after RU486 treatment, and then treated with TPA at 30 µg per mouse twice a week until study endpoint, determined by tumor size of 2 cm in diameter, lesions becoming necrotic or ulcerated, or mice being moribund. Mice were evaluated weekly for papilloma development.

### Apoptosis and proliferation analysis

Apoptosis and proliferation analysis were performed as previously described^31^. For apoptosis, a TdT In Situ Apoptosis Detection Kit (Bio-Techne Corporation, R&D Systems, Cat 4810-30-CK) was used. Briefly, the paraffin slides were de-paraffinized, incubated with TdT enzyme at 37 °C for 1 h, washed in TACS 2 TdT stop buffer for 10 min, incubated with streptavidin-HRP for 30 min, and counterstained with methanol green for 10 min. For proliferation analysis, a BrdU cell proliferation Assay Kit (BioVision, Cat. K306), according to the manufacturer’s protocol.

### BrdU label-retaining cell analysis

Newborn pups were treated with 100 µl of 0.2 µg/µl RU486 for 5 consecutive days. Then they were injected with 20 µl of 12.5 mg/ml BrdU every 12 h for a total of 4 injections starting at 10-days old. Skin sections were collected at 75 days after injection and fixed in 10% formalin-neutral buffer. Samples were embedded in paraffin. BrdU retaining cells were detected using immunofluorescence with rat anti-BrdU. BrdU labeled cells were counted from three tissue sections from each mouse skin.

### Culture of rapidly adherent epidermal cells and colony-forming assay

Newborn pups were treated with 20 µg RU486 for 3 consecutive days. Primary neonatal murine keratinocytes were then isolated from day 4 pups as described^68^. Keratinocytes were plated in the mouse keratinocyte CnT-07 medium (CELLnTEC) at a density of 3 × 10^5^ cells for 10 min at room temperature on dishes coated with collagen type IV. The nonadherent cells were then rinsed off, and after 10 days the growth of rapidly adherent cells was assessed for colony formation by staining with crystal violet for 20 min, and counting colonies. Pictures were acquired on Leica DMIRE2 microscopy.

## Supplementary information

Supplemental information
