# Supplementary material for: Altering MYC phosphorylation in the epidermis increases the stem cell population and contributes to the development, progression, and metastasis of squamous cell carcinoma
Source: Oncogenesis. 2020 Sep 7;9(9):79. doi: 10.1038/s41389-020-00261-3 (PMC7477541; doi:10.1038/s41389-020-00261-3)
Supplement: Supplementary file 1 — Supplemental information [file 41389_2020_261_MOESM1_ESM.pdf]

Wang, et al.
Supplemental Figure Legends:

Supplemental Figure 1

A. Representative images showing ectopic MYC expression by anti-HA staining in control, MYC^{WT}, and MYC^{T58A} mice in hyperplasia and papilloma tissues after DMBA/TPA treatment. **B.** Endogenous *Myc* mRNA expression was suppressed in MYC^{WT} and MYC^{T58A} mice compared to the control mice, as measured by quantitative RT-PCR. p-value is from a one-way ANOVA, followed by a Tukey's multiple comparison test, *** indicates $p<0.001$, ns = no significance. **C**. Quantitative RT-PCR for total *Myc* mRNA expression in epidermal lesions from DMBA/TPA treated mice. MYC^{WT} and MYC^{T58A} mice showed on average a two-fold increase in *Myc* expression compared to the control mice. Significance was determined by one-way ANOVA, followed by a Tukey's multiple comparison test, * indicates $p<0.05$, ns= no significance. **D.** pS62-MYC expression in epidermal hyperplasia of MYC^{WT} or MYC^{T58A} mice as compared to control mice after DMBA/TPA treatment.

Supplemental Figure 2

A, B. Representative images of staining for pS62-MYC, pT58-MYC, or total MYC (Y69 antibody) in SCC of the head and neck. Shown is floor mouth SCC (A) and tongue SCC (B) as well as their respective adjacent mucosa. **C.** Oncoprints from cBioPortal showing the percent of human skin malignancies in the TCGA database with changes in *MYC* gene amplification and/or mRNA upregulation (>2 fold) (cBioPortal). 279 human head and neck squamous cell carcinoma (HNSCC) samples were included in this analysis. **D**. Oncoprints from cBioPortal showing 530 human HNSCC samples (provisional dataset). Green bars in the top 4 rows indicate whether a given sample was profiled for mutations, protein expression (by RPPA), putative copy-number alterations (from GISTIC), or for mRNA expression (RNA Seq V2 RSEM), respectively. Colored bars in bottom four rows indicate presence of alterations as shown for MYC, SOX2, LGR6, and LIN28B. **E.** Table showing co-occurrence of alterations in the four genes.

Wang Supplemental Figure S1

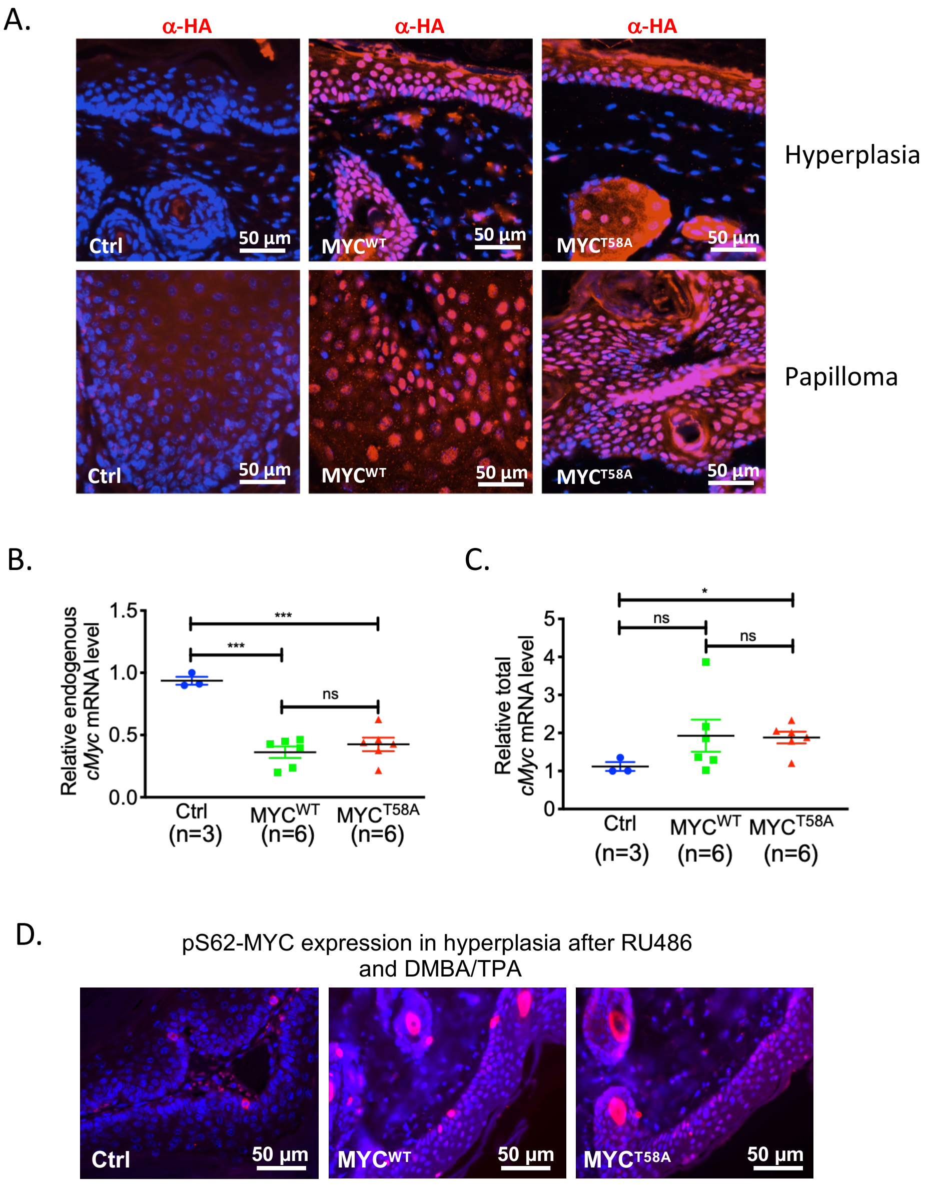

Wang Supplemental Figure S2

A.

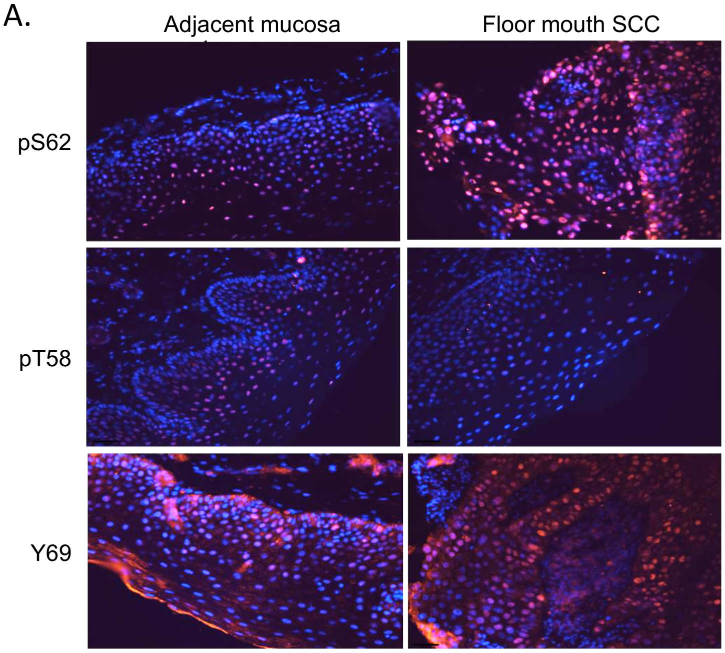

B.

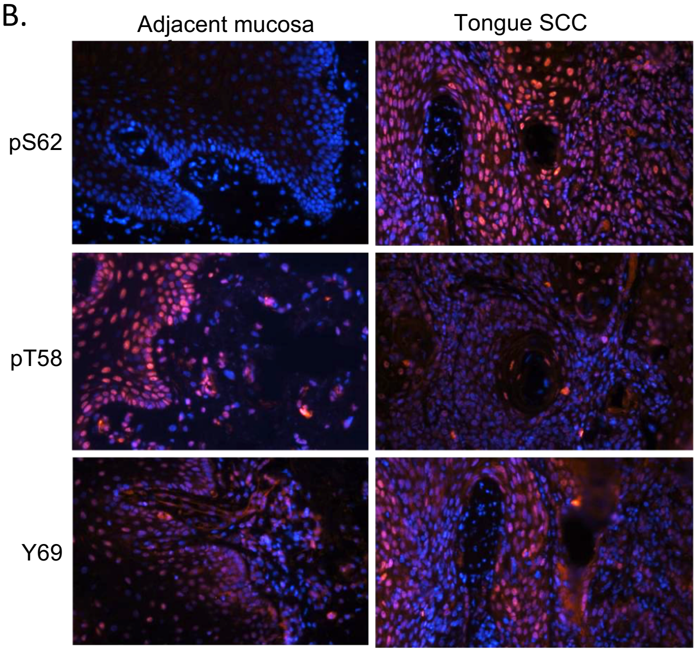

C.

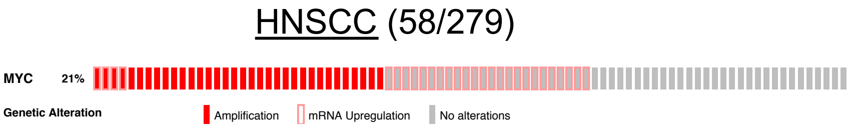

D.

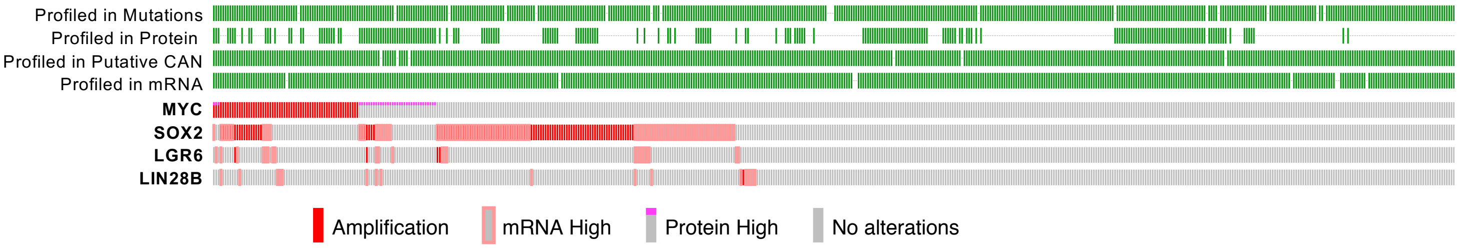

E.

A	B	Neither	A Not B	B Not A	Both	Log2 Odds Ratio	p-Value	q-Value	Tendency
SOX2: AMP EXP>2	LGR6: AMP EXP>2	359	144	6	21	>3	<0.001	<0.001	Co-occurrence
MYC: AMP EXP>2 PROT>1	LGR6: AMP EXP>2	421	82	14	13	2.253	<0.001	<0.001	Co-occurrence
MYC: AMP EXP>2 PROT>1	LIN28B: AMP EXP>2	425	87	10	8	1.966	0.007	0.015	Co-occurrence
LGR6: AMP EXP>2	LIN28B: AMP EXP>2	489	23	14	4	2.603	0.01	0.015	Co-occurrence
MYC: AMP EXP>2 PROT>1	SOX2: AMP EXP>2	307	58	128	37	0.614	0.047	0.056	Co-occurrence
SOX2: AMP EXP>2	LIN28B: AMP EXP>2	355	157	10	8	0.855	0.162	0.162	Co-occurrence
